# The Three-Step Approach for Mental Health 2017 and Beyond

**DOI:** 10.3389/fpubh.2017.00085

**Published:** 2017-04-24

**Authors:** Yvain Rumalean

**Affiliations:** ^1^Psychiatry, NHS, Hertfordshire, UK; ^2^Forensic Legal Medicine, London Met Police, London, UK; ^3^Specialist Adviser CQC, Newcastle upon Tyne, UK

**Keywords:** public policy, public mental health, psychiatry, diagnosis, differential, overdiagnosis

We will need to consider a radically different approach in providing support for people with mental health difficulties. In reality, there is a mismatch between guidelines in diagnosis and management of most mental illness, with realistic implementation of care plans especially when resources are scarce. Despite organizations’ motivation for staff to be creative, we have not been able to optimize on the use of limited resources effectively.

First, guidelines on diagnosis and intervention in mental illness should be considered as just exactly that, guidelines, due to what we know about mental health being heterogeneous in its nature and due to the many fluid, unquantifiable, factors, which influence mental health. Escorpizo et al. (1) stated that “there is no standard platform in which the disease and its impact on functioning are concurrently used within an integrated health information system. Efforts to capture the impact of a disease in a structured and systematic way have so far been hampered by the failure to link the ICD and the ICF at a conceptual and operational level” (p. 1471). They discussed that “the operationalization of integrated disease-and functioning models currently varies, is fragmented across healthcare settings, and is perhaps more commonly observed in healthcare systems with medium to advanced infrastructures and access to resources” (p. 1472, 1474). Following the abundance of knowledge so far, based on very important studies already carried out in Psychiatry, Psychology, Sociology, and other related specialties, we should now try to use the existing knowledge in a novel way and not be afraid to be more tangential from guidelines as long as the approach is in-keeping with patient care being consistently central and with considered risk assessments.

Second, there should be more leadership in promoting patients’ autonomy especially toward contribution in recovery and more importantly in promoting preventative work, in preventing patients from becoming disproportionately dependent on services. This could be achieved by ensuring that overmedicalization/overdiagnosis is significantly reduced especially for those who have traits of personality disorder and drugs/alcohol misuse, to name only a few. When overmedicalization is minimized, patients’ autonomy is upheld in encouraging patients to actively contribute toward their own prevention and recovery, and for patients not to be too quick to blame others or any potential mental illness diagnosis. In mental health diagnosis, a categorical approach “contrasts with clinical medicine where the clinical significance of subthreshold symptoms is well recognized” and “is often criticized for being rigid and reductionist in practice” (p. 340) as discussed by Carragher et al. (2). They went on to say that “polythetic-categorical approach gives rise to significant heterogeneity within diagnostic groupings as it does not account for differences in clinical presentation” (p. 340).

Third, there needs to be an honest motivation in staff at all levels within any organizations that provide support for individuals who (provisionally) have mental health difficulties; far too often, dysfunctional teams are led by inexperienced senior staff due to understaffing and premature promotions, pressured by demands of “meeting (financial) targets.” The Health of the Nation Outcome Scales’ 12 scales (NHS’ HoNOS) are used by clinical staff on service users with severe mental illness “for several health and social functioning variables” (p. 116) mentioned by Speak et al. (3). They explained that “payment by results (PbR) represents a significant change in the way mental health services will be commissioned and funded in England … England has therefore progressed PbR implementation for mental health more than other countries. In the outcomes and quality framework for England’s new mental health PbR system, several indicators and outcome measures are to be introduced; one is the Health of the Nations Outcome Scales (HoNOS) which is widely used tool for evaluating mental health service outcomes” (p. 115–6). Speak et al. (3) raised issues with HoNOS which “are relevant to all countries in which it is used as an outcome measure; particularly if HoNOS is used to report national outcomes or is considered for use within mental health PbR systems” (p. 126).

The bulk of service provision should be about being aware of service limitation, psychoeducation for, and about improving confidence in staff, students, patients, and their support network, most of which might not include active treatment, which in turn, could be more cost-effective. Furthermore, this could improve upon staff recruitment, retention, and patients/carers’ satisfaction. This work ethos would most likely help with complex policy process; policy process specifically in mental health proves to be extremely challenging. This partly stems from “the problem that no one owns health (and hence no one can be mobilized for its advocacy) cannot simply be attributed to a lack of resource or political will in the (public) policy-making environment, but seems reciprocally unsupported by a similar lack of commitment among the public” (p. 161) as discussed by de Leeuw et al. (4). In mental health, this problem is maintained historically by and as an ongoing issue of stigma.

Service provision should be decentralized; service provision should be unique to specific areas with their own demographics and own patients as different areas have different prevalence and incidence of mental illness. Policy process would need to strive toward one that fits most and not one that fits all. This approach could lead to a more accurate application/utilization of funding.

Kirmayer (5) raised an important issue that “strong interconnections of values framed at one level with those at other levels means that there are likely to be unavoidable tradeoffs between different values or desirable short- and long-term outcomes such as energy, efficiency, happiness, maturation, depth of personality, and responsiveness to social and moral predicaments. These tradeoffs challenge the assumption of universalism in biomedicine and raise questions about the consequences of our willingness to use medications to treat the myriad forms of distress that may signal fundamental problems with our way of life” (p. 295).

Bearing Kirmayer’s vital viewpoint in mind, service provision which is decentralized, unique to geographical areas, and driven by honest staff motivation with appropriate training and confident senior staff could lead to a delivery of high-quality service for patients where staff and patients are both held accountable.

## Author Contributions

YR is a medical doctor who has worked for the National Health Service, UK, since 1997. He is a Psychiatrist for Hertfordshire Partnership University Foundation NHS Trust and a Forensic Medical Examiner for the London Metropolitan Police. He has worked with colleagues to attend to complex interface issues within and between service providers and was an adviser for NHS’ National Clinical Assessment Service. He is a specialist adviser for Care Quality Commission (CQC) UK, and a reviewer of WHO’s training package on Human Rights and Mental Health.

## Conflict of Interest Statement

The author declares that the research was conducted in the absence of any commercial or financial relationships that could be construed as a potential conflict of interest.
